# Supplementary material for: TxCyto: A machine learning framework for estimating cytokine activity from whole transcriptome
Source: bioRxiv. 2026 Sep 29:2026.09.28.754405. Preprint. [Version 1] doi: 10.64898/2026.09.28.754405 (PMC13648652; doi:10.64898/2026.09.28.754405)
Supplement: Supplementary file 1 [file NIHPP2026.09.28.754405v1-supplement-1.pdf]

bioRxiv preprint doi: https://doi.org/10.64898/2026.09.28.754405; this version posted September 29, 2026. The copyright holder for this preprint (which was not certified by peer review) is the author/funder. This article is a US Government work. It is not subject to copyright under 17 USC 105 and is also made available for use under a CC0 license.

## Supplementary Figures

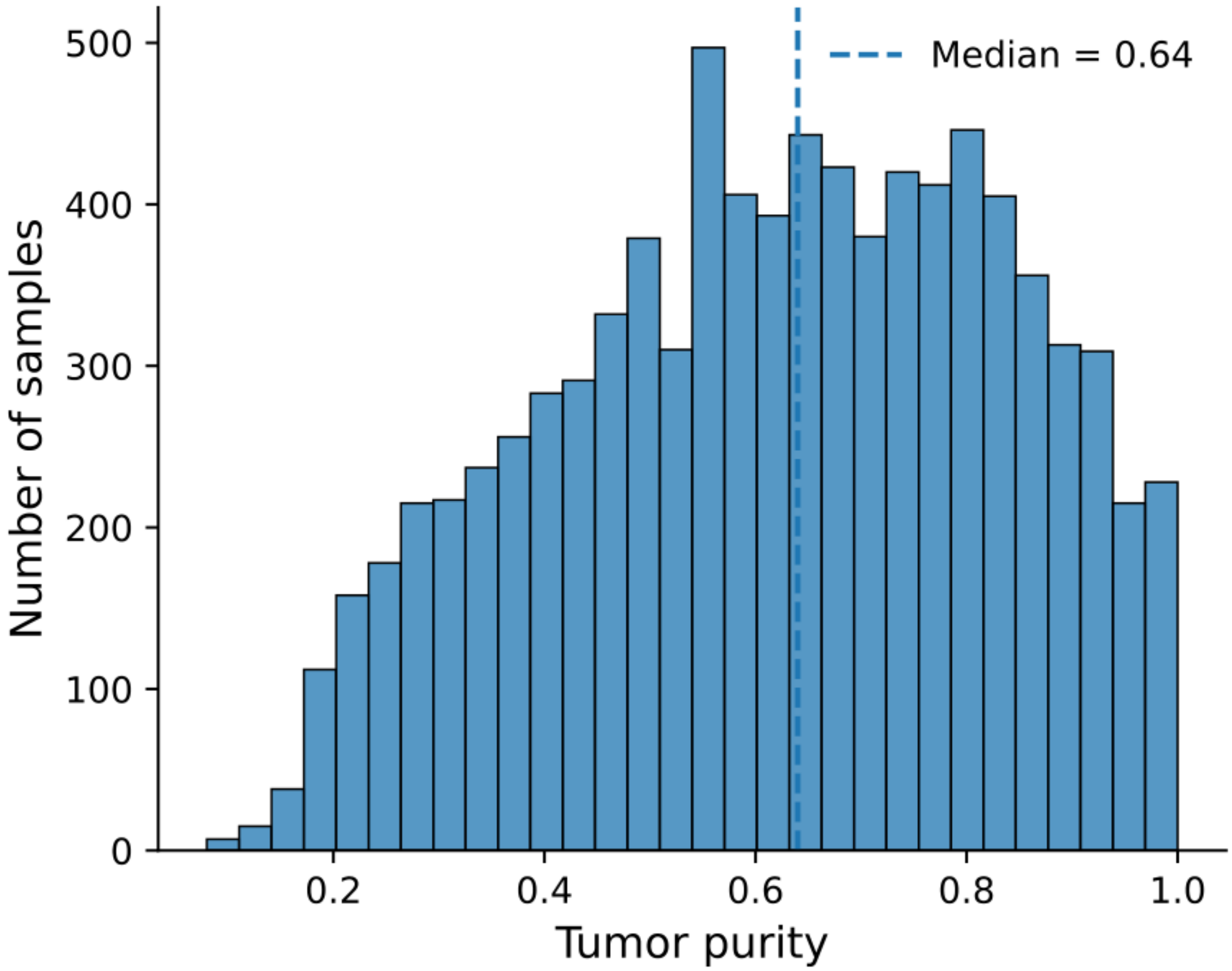


**Supplementary Figure 1.** The distribution of the purity score of TCGA tumor samples having median purity score~0.65.

bioRxiv preprint doi: https://doi.org/10.64898/2026.09.28.754405; this version posted September 29, 2026. The copyright holder for this preprint (which was not certified by peer review) is the author/funder. This article is a US Government work. It is not subject to copyright under 17 USC 105 and is also made available for use under a CC0 license.

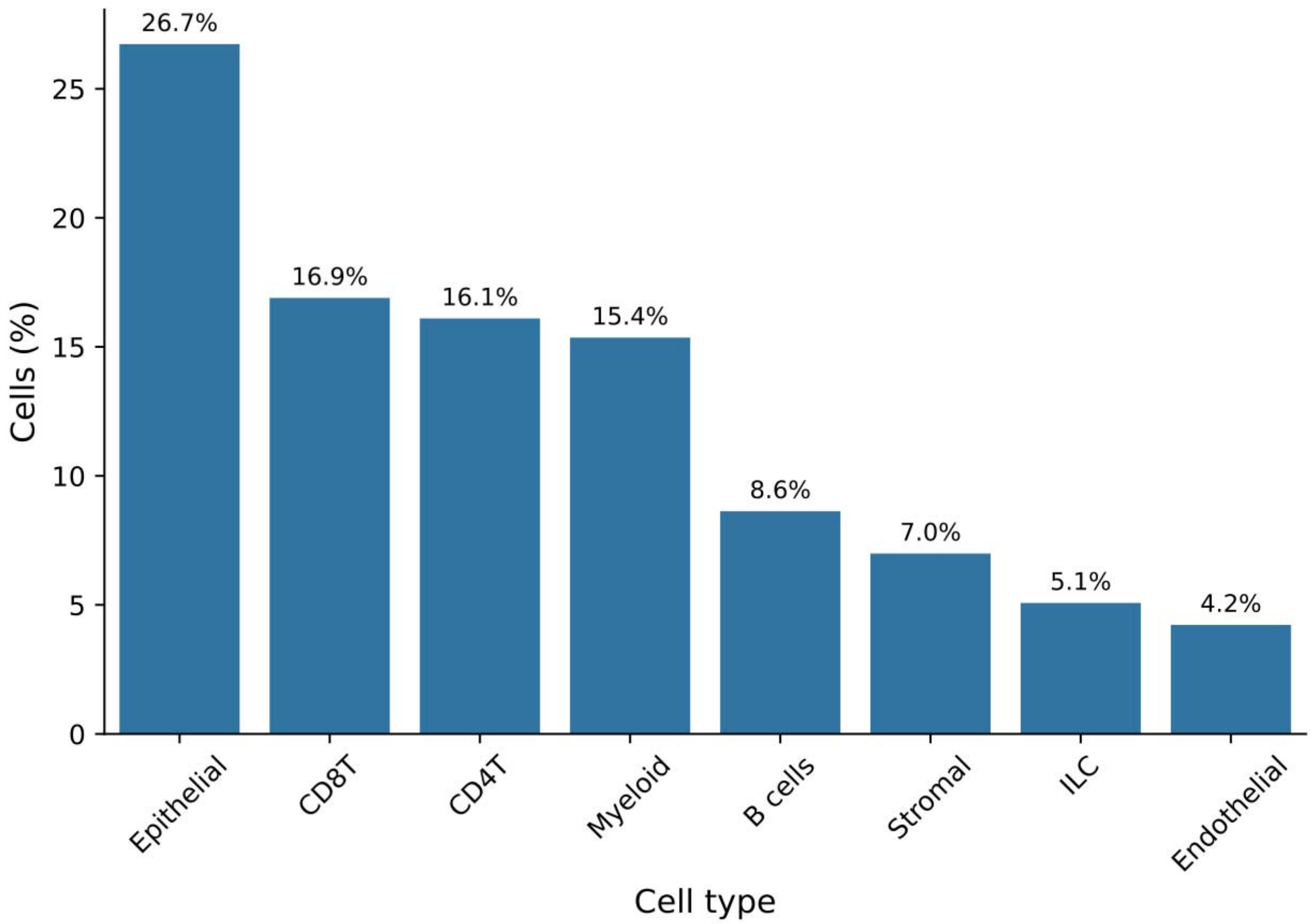


**Supplementary Figure 2.** The plot shows the proportion of different cell types across 1062 single-cell RNA-seq samples.

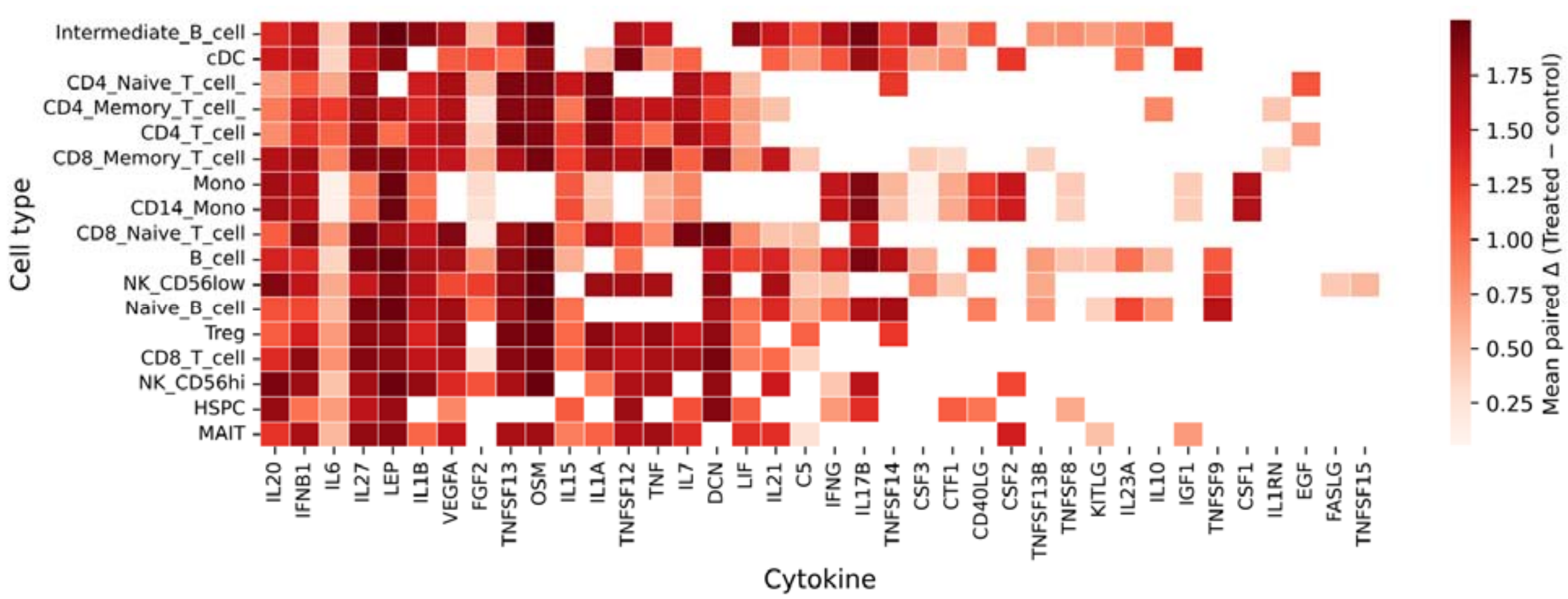


**Supplementary Figure 3.** The heatmap shows the significantly elevated cytokines (p-value <0.05) across different immune cell populations of treated PBMC samples.
